# Predictive Factors of Physicians’ Satisfaction and Quality of Work Under Teleconsultation Conditions: Structural Equation Analysis

**DOI:** 10.2196/47810

**Published:** 2024-06-10

**Authors:** Liliana Hawrysz, Magdalena Kludacz-Alessandri, Renata Walczak

**Affiliations:** 1 Faculty of Management Wrocław University of Science and Technology Wroclaw Poland; 2 College of Economics and Social Sciences Warsaw University of Technology Plock Poland; 3 Faculty of Civil Engineering, Mechanics and Petrochemistry Warsaw University of Technology Plock Poland

**Keywords:** perceived ease of use, perceived usefulness, physicians’ satisfaction, behavioral intention to use telemedicine, health care quality, technology acceptance model, TAM, COVID-19: telemedicine

## Abstract

**Background:**

The COVID-19 pandemic contributed to an increase in teleconsultation adoption in the Polish primary health care system. It is expected that in the long run, teleconsultations will successfully replace a significant part of face-to-face visits. Therefore, a significant challenge facing primary health care facilities (PHCs) is the acceptance of teleconsultations by their users, especially physicians.

**Objective:**

This study aimed to explore physicians’ acceptance of teleconsultations during the COVID-19 pandemic in Poland.

**Methods:**

A representative survey was conducted among 361 physicians of PHCs across Poland in 2021. For the purposes of the study, we developed a modified Technology Acceptance Model (TAM) model. Based on the modified TAM, we analyzed the impact of perceived usefulness (PU), perceived ease of use (PEU), and intention to use teleconsultation (INT) on physicians’ satisfaction (SAT) and quality of work (Q). The psychometric properties of the research instrument were examined using exploratory factor analysis. Finally, structural equation modeling was used for data analysis.

**Results:**

The results indicated a generally high level of PU (mean 3.85-4.36, SD 0.87-1.18), PEU (mean 3.81-4.60, SD 0.60-1.42), INT (mean 3.87-4.22, SD 0.89-1.12), and SAT (mean 3.55-4.13, SD 0.88-1.16); the lowest rated dimension in TAM was Q (mean 3.28-3.73, SD 1.06-1.26). The most important independent variable was PU. The influence of PU on INT (estimate=0.63, critical ratio [CR]=15.84, *P*<.001) and of PU on SAT (estimate=0.44, CR= 9.53, *P*<.001) was strong. INT was also a key factor influencing SAT (estimate=0.4, CR=8.57, *P*<.001). A weaker relationship was noted in the effect of PEU on INT (estimate=0.17, CR=4.31, *P*<.001). In turn, Q was positively influenced by INT (estimate=0.179, CR=3.64, *P*<.001), PU (estimate=0.246, CR=4.79, *P*<.001), PEU (estimate=0.18, CR=4.93, *P*<.001), and SAT (estimate=0.357, CR=6.97, *P*<.001). All paths between the constructs (PU, PEU, INT, SAT, and Q) were statistically significant, which highlights the multifaceted nature of the adoption of teleconsultations among physicians.

**Conclusions:**

Our findings provide strong empirical support for the hypothesized relationships in TAM. The findings suggest that the PU and PEU of teleconsultation have a significant impact on the intention of physicians to adopt teleconsultation. This results in an improvement in the satisfaction of Polish physicians with the use of teleconsultation and an increase in Q. The study contributes to both theory and practice by identifying important prognostic factors affecting physicians’ acceptance of teleconsultation systems.

## Introduction

### Background

Telemedicine is an IT-based method that has the potential to support and enhance physicians’ patient care [1]. Telemedicine is defined as a tool using information and communication technology (ICT) that is used to support and promote remote care, health-related professional education, and public health administration [2]. One of the basic forms of telemedicine is teleconsultations. Medical consultations have been provided remotely (teleconsultations), by telephone, or by instant messaging for many years, but the COVID-19 pandemic has forced widespread use of this form of communication with patients. Teleconsultations have reduced the high costs of medical services and the queues of patients in clinics [3]. In Poland, the need for teleconsultations began at the beginning of the pandemic. Most physicians had to adapt to the requirements of COVID-19 rules and regulations. Even though the need for teleconsultations has already been officially abolished, telemedicine is still the future of medicine. Not all advice has to be given to patients on-site in a clinic. In the face of a decreasing number of physicians, an aging society, and an increased demand for health care, telemedicine seems to be a solution that will solve the problems of personnel shortage. However, the use of telemedicine tools requires the medical staff to be proficient in using them and to accept and support such solutions.

The aim of our study was to examine the physicians’ satisfaction (SAT) and quality of work (Q) in conditions of teleconsultations during the COVID-19 pandemic in Poland. For the purposes of the study, we developed a modified Technology Acceptance Model (TAM) and tested it in primary health care facilities (PHCs) in Poland. This model allowed us to analyze the impact of perceived usefulness (PU), perceived ease of use (PEU), and intention to use teleconsultation (INT) on SAT and Q. In our study, we focused primarily on teleconsultations, consisting of telephone and video conversations between physicians and patients [2]. Teleconsultations are the basic form of telemedicine in Poland, and during the COVID-19 pandemic, they were the only form of PHC in the country [4].

The first part of this paper analyzes the literature focusing on TAM in relation to the research hypotheses. The second part contains a description of the research methodology used in this study, with emphasis on the validation of the research tool developed for measuring the modified elements of TAM. The following section presents the results regarding the analyzed constructs of the model and the results of structural equation modeling (SEM). The last part of the paper contains the discussion, conclusions and practical implications.

### Literature Review

Until March 2020, the use of telemedicine was negligible and mainly concerned patients in rural areas. In 2019, teleconsultations accounted for as much as 8% of all medical visits in the United States [5]. The situation has changed significantly after the outbreak of the COVID-19 pandemic, especially in the case of emergency visits. In the United States, there was a 683% increase in teleconsultations between March 2 and April 14, 2020 [5]. Researchers agree that the COVID-19 pandemic has changed the way we think about telemedicine. First and foremost, it popularized this way of providing medical services. However, its primary advantage is to protect both patients and physicians from the risk of virus infection [6,7]. In the long term, researchers believe that telemedicine can successfully replace a significant portion of face-to-face (F2F) visits; however, it will not eliminate them completely [4,8,9].

Telemedicine is not a new concept. Decades ago, pioneering projects emerged to test the concept of telemedicine or evaluate its applicability. However, most of these telemedicine projects failed to meet expectations. The failure was blamed on an underdeveloped and mostly primitive IT infrastructure, immature technology, and ineffective use [1]. The failure of first-generation telemedicine projects prompted an in-depth analysis and rigorous assessment of the technological, social, cultural, and organizational dimensions surrounding their introduction.

Resistance from users of new technologies in the medical community is as natural as possible, even if users are aware of the benefits this technology brings with it [10]. Therefore, an essential organizational challenge facing health care organizations considering or planning to provide telemedicine-enabled health care services is technology acceptance by users [1]. The problem regarding acceptance of technology has been discussed for a long time [11], and many models have been developed to assess users’ attitudes toward new solutions [12-15]. Acceptance of ICT by physicians providing health care has also been assessed [16-18].

TAM is the most popular model in the literature for testing the acceptance of technology [19]. Van Schaik et al [20] used TAM to assess the attitudes of physiotherapists toward new medical technologies. Chau and Hu [21] studied the acceptance of telemedicine technologies by physicians. Holden and Karsh [22] extensively reviewed the literature on TAM applications and related models for ICT acceptance by health care professionals and, more specifically, health care information systems. They noted that in health care, there is a need for a complete approach to technology acceptance testing than for other professionals from companies or ICT organizations [22]. We chose to use TAM in our study because it is general, parsimonious, and ICT specific. It is designed to provide an explanation and prediction of the acceptance of a wide range of ICTs by a diverse population of users in different organizational contexts. In addition, the model has a well-researched and validated list of psychometric measurements, and this makes its use operationally attractive. Finally, TAM is the dominant model for studying user acceptance of technology, and over the years, it has accumulated satisfactory empirical support for its overall explanatory power and assumed individual causal relationships [1,2]. Over the past few decades, many researchers have proven that TAM enriched with certain other constructs is better suited to research and explain the acceptance of new information technologies by users [23].

TAM analyzes the influence of various factors on the intention to use new technology, among which the main role is played by PU and PEU [24]. PU is defined as the extent to which the user’s work performance is expected to improve through the use of new technology [2,25]. Similarly, Davis [11] defined PU as an individual’s perceptions regarding the outcome of the experience with technology. In the area of health care, Kissi et al [26] defined PU as “physicians’ belief regarding the benefits of telemedicine services that they improve access to medical care, the flow of medical records and patients’ health.”

PEU, in contrast, is the degree to which using new technology is expected to be effortless [2,25,27]. In the area of health care, PEU describes how physicians perceive telemedicine services in terms of their ease of use and learning [26].

TAM suggests that actual technology usage is determined by individuals’ INT [2]. INT is understood as a motivation encouraging the system’s user to use the system continuously, and in the case of physicians, it concerns their motivation to use telemedicine services, including, above all, teleconsultations [26]. INT is affected by PU, PEU, and users’ attitudes toward technology [2,19]. Lin et al [28] used an integrated approach with the key elements from TAM and assessed the technology acceptance by health professionals of what they called “personal digital assistance (PDA).” The main variables from TAM (PU and self-efficacy) determined INT [28]. Similar conclusions were reached by Zayyad and Toycan [29], Chau and Hu [30], Tubaishat [31], and Vitari and Ologeanu-Taddei [32]. Thus, our first research hypotheses were proposed as follows:

- Hypothesis (H)1: PU has a direct effect on INT.
- H2: PEU has a direct effect on INT.

The results of Lin et al [28] showed that the traditional variables of TAM can be effectively integrated with variables from other theoretical approaches, which may help better understand the acceptance of new technologies by health care professionals [33]. There are relatively fewer TAM tests and modifications in the health care sector. Therefore, it is worth making such attempts to broaden the knowledge about new factors affecting physicians’ acceptance of technologies. In our research, we decided to include 2 new constructs: SAT and Q. SAT explains how satisfied users are with using a particular service [19]; Q explains how physicians assess the value and worth of their work with the use of teleconsultations. The addition of these constructs was a consequence of both literature reviews and interviews with physicians (pilot survey). Bhattacherjee and Premkumar [10,34-36] noted that in the use phase of technology (postacceptance), PU is positively associated with user SAT. Alsohime et al [37] also confirmed the effect of PU on SAT, noting the significant impact of training courses before the implementation of new technology. Petter and Fruhling [38] confirmed that the SAT that users have with the information system positively affects their INT. As a consequence, we presented the subsequent hypotheses:

- H3: PU has a direct effect on SAT.
- H4: INT has a direct effect on SAT.

In our previous studies examining technology acceptance in Polish PHC facilities, we developed a conceptual framework defining the impact of PU and PEU on the need for teleconsultation adoption and examined the influence of selected behavioral factors on these constructs [2]. In this paper, we enriched these previous studies by analyzing the impact of PU and PEU as independent variables on SAT and Q. In our analysis, we additionally considered INT as a mediating variable in the model. This is the first study that extends TAM to include an analysis of SAT and Q in the teleconsultation condition.

Padilha et al [39] surveyed students and nurses’ ease, usefulness, and intention to use a Massive Open Online Course. Findings confirmed the significant impact of PU, PEU, and INT on the current and future Q the groups studied [39]. Similar conclusions were reached by Saputra et al [40] and Chirchir et al [41]. Souza et al [42] proposed a process model for the evaluation of the Q of clinical decision support systems following the ISO/IEC 25022 and ISO/IEC 25010 standards, part of which was to identify the effect of SAT on Q. Given these considerations, we proposed the following hypotheses:

- H5: PU has a direct effect on Q.
- H6: PEU has a direct effect on Q.
- H7: INT has a direct effect on Q.
- H8: SAT has a direct effect on Q.

Although according to the research conducted so far, TAM is a reliable model for examining technology acceptance in PHC facilities, we can always try to supplement it with new research constructs [2]. TAM, in our study, was supplemented with 2 constructs, SAT and Q, which will contribute to the health care literature.

The Polish health care system is based on an insurance model. PHC physicians in Poland must be health insurance physicians who have a contract with the National Health Fund (NHF) to provide health care services. The functioning of PHCs in Poland is based on the right of patients to personally choose a preferred physician. The selected physicians receive an annual capitation fee for each registered patient [43]. PHC facilities in Poland function as both state-owned and private facilities. Both sign contracts to provide services that are free to the patient and paid for by the NHF. Each facility is managed according to its own rules. Private facilities have more flexibility in making decisions and in hiring and paying employees. Public facilities are subject to top-down regulations governing their operations. As private facilities provide fee-based services, in addition to free services, they have more resources to pay salaries and run their operations.

## Methods

### Data Collection

In this cross-sectional study, the survey followed a multimodal approach, integrating computer-assisted web interviewing (CAWI), computer-assisted telephone interviewing (CATI), and paper-and-pencil interviewing (PAPI) techniques across a statistically representative sample of 371 PHC facilities. This number was derived from a total of 5503 outpatient PHC facilities in Poland, calculated to be representative at a 95% CI level, with a 50% response distribution and a 5% margin of error, for the aforementioned assumptions, and the minimum survey sample size was 359 [44-47]. The survey sample was randomly selected from the BISNODE database, which includes comprehensive information on all Polish PHC facilities. Of 5503 outpatient facilities in Poland, 371 (6.7%) were successfully surveyed, with each representing 1 physician providing remote medical advice. The survey process entailed replacing nonparticipating facilities with other randomly chosen facilities, ensuring the integrity and representativeness of the sample. Quality control measures were rigorously followed, with a certified polling company overseeing the survey execution. Instances of schematic responses and unusually short survey durations led to the exclusion and replacement of certain responses, resulting in a final analytical sample of 361 (97.3%) records. Before filling the questionnaire, the physicians were informed that the questionnaire is aimed at PHC physicians and concerns the evaluation of their satisfaction with the use of the teleconsultation system for the provision of patient care.

The sample was limited to 1 PHC physician from each randomly selected facility in Poland. This approach was adopted for several reasons. Conducting a survey that included multiple physicians from each facility would have significantly increased the scale and complexity of the study. Given resource constraints, such as funding, time, and personnel, it was more feasible to limit the number of participants, while still achieving a representative sample. The aim was to obtain a broad overview of the acceptance and satisfaction with teleconsultation across a wide range of PHC facilities in Poland. By selecting 1 physician from each facility, we ensured a diverse and statistically representative sample of the entire population of PHC facilities, which may not have been possible with a more concentrated sample from fewer facilities. The study was primarily designed to assess the impact of system-level factors (eg, PU and PUE) on the acceptance of telemedicine. Although individual characteristics, such as age and gender, are important, the primary focus was on broader systemic issues that could be generalized across the population. Conducting an extensive survey during the COVID-19 pandemic posed unique challenges, including limited access to PHC facilities and the need to minimize contact. The study design did not include a detailed examination of individual physician factors and their impact on the acceptance of telemedicine, and the approach was strategically chosen to balance comprehensiveness, feasibility, and the overarching research objectives.

### Survey Instrument and Measures

The survey instrument contained 2 groups of statements and questions: statements about analyzed latent factors and general questions about age and gender of the respondent, legal status of the PHC facility, voluntariness of providing remote advice, and ways in which the respondent provided remote advice. The questionnaire included 48 statements and questions, but only the statements used in the modified TAM are presented in Multimedia Appendix 1. A 5-point Likert scale was used in this study: 1 (I do not agree), 2 (I do not agree somewhat), 3 (I neither agree nor disagree), 4 (I agree somewhat), and 5 (I agree).

SEM in this study was adapted from the original TAM, which identifies PU and PEU as the principal determinants of technology use and acceptance. The PU variable measures the degree to which physicians believe that teleconsultations improve their work efficiency and patient care. It encompasses aspects such as enhanced health care delivery, better documentation, and cost-effective monitoring. PU in this context is gauged through 6 survey statements (PU1-PU6), which assess various dimensions of utility that teleconsultations provide in the health care setting [11,48-51]. PEU variables are defined as the extent to which physicians believe that teleconsultations are effortless to learn and implement. The variables relate to the ease of use of the teleconsultation system and the use of medical data. This construct is evaluated via 7 survey statements (PEU1-PEU7), focusing on the usability and accessibility of the teleconsultation system [2,25,27]. INT, similar to that in TAM, used as a mediating variable, represents the likelihood of physicians continuing to use teleconsultations in the future. It is measured through 5 survey statements (INT1-INT5), focusing on the perceived long-term utility and effectiveness of teleconsultations in patient diagnosis and care [11,12,41]. Q, used as an independent variable, refers to the perceived enhancement in work value and worth due to teleconsultations and is gauged through 3 survey statements (Q1-Q3). It assesses whether teleconsultations uphold the standard of traditional visits and enable comprehensive patient care [22]. The SAT variable measures the overall contentment of physicians with teleconsultations. It includes aspects such as convenience compared to traditional visits and comfort in providing remote advice [11,52] and is assessed through 4 survey statements (SAT1-SAT4) [12,13,53,54].

### Exploratory Factor Analysis

The study required validating the structure and dynamics within the adapted TAM, because the original model was extended to encompass SAT and Q. The evaluation of the survey statements for inclusion in the factors measuring the assessed dimensions was based on exploratory factor analysis (EFA). EFA was used to select the final variables for the structural model. For each dimension, EFA was separately carried out to assess the Kaiser-Meyer-Olkin (KMO) measure of sampling adequacy. The value of this index should be >0.7 [55]. For all dimensions, this condition was fulfilled, but the KMO value of the PEU dimension was <0.7. The EFA results for each dimension are presented in Table 1.

In addition, the ability of each dimension to be represented by individual survey statements was assessed using Bartlett’s test of sphericity. For each dimension, the chi-square value was significant, and in each case, *P*<.001. Based on EFA, the PEU dimension was finally divided into 2 separate factors: PEU_1 and PEU_2. Questions PEU1, PEU2, PEU3, and PEU4 were about the technical ease of use of the system (PEU_1), and questions PEU5, PEU6, and PEU7 were about the ease of use of the system from the point of view of handling medical data (PEU_2). Reliability analysis was conducted for all dimensions of the validity of using the adopted statements to measure each factor. Cronbach coefficients were determined for each factor, with acceptable values falling within the range of 0.7-0.95 [56]. The constructs were confirmed to possess suitable psychometric properties, enabling their effective use in SEM analysis. For the PEU dimension, reliability analysis did not give a clear answer as to which statement should be removed to improve the Cronbach and KMO coefficient values. The use of survey statements to measure the PEU dimension requires confirmation in the structural model. The EFA results for the PEU dimension are presented in Table 2, and component factor loadings are presented in Table 3.

**Figure 1:**
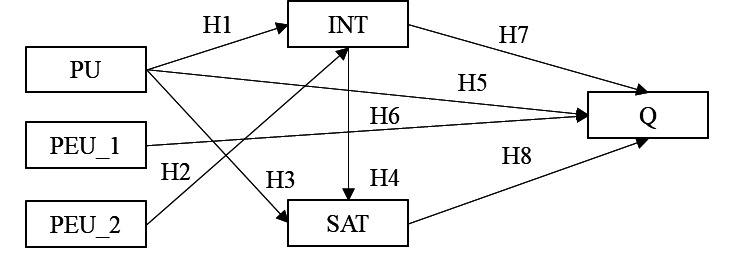
Structural model depicting the factors affecting SAT with teleconsultation and Q. Research hypotheses H1-H8 for direct paths. H: hypothesis; INT: intention to use teleconsultation; PEU: perceived ease of use; PU: perceived usefulness; Q: quality of work; SAT: satisfaction.

Based on imputed factors, a structural model was prepared, where the effects of PU, PEU_1, and PEU_2 on INT, SAT, and Q were studied. Figure 1 shows the final tested model with only significant dependencies.

**Table 1:** Component factor loadings.^a^

Variable	Factor					
	PU^b^	PEU^c^_1	PEU_2	INT^d^	SAT^e^	Q^f^
KMO^g^	0.90	0.69	0.69	0.80	0.77	0.73
Cronbach α	0.89	0.71	0.71	0.81	0..89	0.86
PU1	0.69	—^h^	—	—	—	—
PU2	0.84	—	—	—	—	—
PU3	0.89	—	—	—	—	—
PU4	0.87	—	—	—	—	—
PU5	0.79	—	—	—	—	—
PU6	0.79	—	—	—	—	—
PEU1	—	0.79	—	—	—	—
PEU2	—	0.69	—	—	—	—
PEU3	—	0.59	—	—	—	—
PEU4	—	0.81	—	—	—	—
PEU5	—	—	0.67	—	—	—
PEU6	—	—	0.86	—	—	—
PEU7	—	—	0.78	—	—	—
INT1	—	—	—	0.80	—	—
INT3	—	—	—	0.82	—	—
INT4	—	—	—	0.76	—	—
INT5	—	—	—	0.83	—	—
SAT1	—	—	—	—	0.78	—
SAT2	—	—	—	—	0.88	—
SAT3	—	—	—	—	0.89	—
SAT4	—	—	—	—	0.82	—
Q1	—	—	—	—	—	0.86
Q2	—	—	—	—	—	0.90
Q3	—	—	—	—	—	0.90

^a^Extraction method: principal component analysis; rotation method: varimax with Kaiser normalization.

^b^PU: perceived usefulness.

^c^PEU: perceived ease of use.

^d^INT: intention to use teleconsultation.

^e^SAT: satisfaction.

^f^Q: quality of work.

^g^KMO: Kaiser-Meyer-Olkin.

^h^Not applicable.

**Table 2:** Total variance of the PEU^a^ dimension explained by EFA^b^.^c^

Component	Initial eigenvalues				Extraction sums of squared loadings				Rotation sums of squared loadings		
	Total	% of Variance	Cumulative %	Total		% of Variance	Cumulative %	Total		% of Variance	Cumulative %
1	2.71	38.68	38.68	2.71		38.68	38.68	2.20		31.40	31.40
2	1.40	19.98	58.65	1.40		19.98	58.65	1.91		27.25	58.65
3	0.90	12.86	71.52	—^d^		—	—	—		—	—
4	0.69	9.83	81.35	—		—	—	—		—	—
5	0.58	8.34	89.69	—		—	—	—		—	—
6	0.37	5.28	94.96	—		—	—	—		—	—
7	0.35	5.04	100.00	—		—	—	—		—	—

^a^PEU: perceived ease of use.

^b^EFA: exploratory factor analysis.

^c^Extraction method: principal component analysis.

^d^Not applicable.

**Table 3:** PEU^a^ factors loadings.^b^

Variable	Factor	
	PEU_1	PEU_2
PEU1	0.787	—^c^
PEU2	0.685	—
PEU3	0.589	—
PEU4	0.808	—
PEU5	—	0.670
PEU6	—	0.863
PEU7	—	0.784

^a^PEU: perceived ease of use.

^b^Extraction method: principal component analysis; rotation method: varimax with Kaiser normalization.

^c^Not applicable.

In the original TAM, the actual use of technology is the independent model variable. In this study, physicians were required to conduct teleconsultations, so INT and SAT variables were treated as mediators affecting the dependent variable, Q. Because of this, the effect of PU, PEU1, and PEU_2 variables on Q was also studied indirectly. The research hypotheses H9-H14 regarding indirect effects were proposed as follows:

- H9: PU has an indirect effect on Q through INT.
- H10: PU has an indirect effect on SAT through INT.
- H11: PU has an indirect effect on Q through INT and SAT.
- H12: PEU_2 has an indirect effect on Q through INT.
- H13: PEU_2 has an indirect effect on SAT through INT.
- H14: PEU_2 has an indirect effect on Q through INT and SAT.

### Ethical Considerations

The survey instrument was approved by the Ethics Committee of the Warsaw University of Technology that issued the Certificate of Ethics Approval (certificate dated January 15, 2021). As a result of the contact, 587 physicians provided consent to participate in the study, of which, despite consent, in 216 (36.8%) PHC facilities, the complete set of surveys could not be completed. Respondents were informed about the purpose of the research before starting the survey. They could withdraw from completing the survey at any time. Study data were anonymous and deidentified. No compensation from respondents was taken for the research.

## Results

### Participant Characteristics

**Figure 2:**
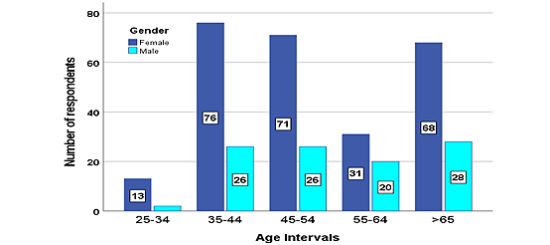
Physicians’ age distribution by gender.

Of the 361 physicians, 199 (55.1%) were in the 35-54–year age group, 94 (26%) were of retirement age and over 65 years old, 260 (72%) were women, and only 101 (28%) were men. The age distribution of the surveyed physicians is presented in Figure 2.

### Evaluation of the Level of TAM Dimensions

The research considered the dimensions originally defined in TAM (PU, PEU, and INT) [11]. Two dimensions were added to the model: SAT and Q.

The PU variable assesses physicians’ perceptions of the utility and benefits of teleconsultation services. The mean scores for PU1-PU6 ranged from 3.85 to 4.36 (SD 0.87-1.18), indicating a generally high level of agreement among physicians that teleconsultations are beneficial to their work. The highest mean score was for PU1 (4.36, SD 0.94), suggesting that the physicians particularly valued teleconsultations during challenging times, such as pandemics. The SDs, ranging from 0.87 (mean 4.24) for PU4 to 1.18 (mean 3.85) for PU3, implied some variability in how the physicians perceived the usefulness of different aspects of teleconsultations. The higher deviation in PU3 indicated more varied opinions about the efficiency enhancement brought by teleconsultations. The skewness for all PU statements was negative, ranging from –0.81 to –1.73, suggesting a tendency among the physicians to agree that teleconsultations are useful in their work. The most pronounced skewness was observed in PU1, indicating strong agreement about the difficulty of work during a pandemic without teleconsultations. The kurtosis values ranged from –0.38 for PU5 to 2.77 for PU1, indicating varied distribution patterns. The higher kurtosis in PU1 reflected a more peaked distribution, suggesting more consistent agreement among physicians regarding its statement. The data suggested that physicians perceive teleconsultations as a valuable tool in their professional practice. The high mean scores across all PU items reflected positive perceptions of the teleconsultation system’s usefulness, particularly in aiding work during a pandemic (PU1). The variation in SDs pointed to some differences in individual opinions about the specific benefits of teleconsultations, such as work efficiency and time-saving aspects. The negative skewness across all items highlighted a general agreement on the usefulness of teleconsultations, with a stronger consensus in areas such as coping with pandemic challenges. The kurtosis values, particularly for PU1, indicated that most responses were concentrated around higher ratings, showing strong agreement in specific areas of usefulness. The PU variable demonstrated that physicians generally regard teleconsultations as a beneficial tool in their practice, particularly under challenging conditions, such as a pandemic. Although there was overall agreement on their utility, the variation in perceptions across different aspects suggests areas where experiences and expectations of teleconsultations may differ among individual physicians.

The PEU variable examines physicians’ perceptions of the ease and effortlessness associated with using teleconsultation systems. The mean scores for PEU1-PEU7 ranged from 3.81 to 4.60 (SD 0.60-1.42), respectively, and indicated a generally high level of agreement among the physicians that teleconsultation systems are user friendly and easy to use. The highest mean score was for PEU7 (4.60, SD 0.60), suggesting that using external systems during teleconsultations was perceived as particularly straightforward by most of the physicians. The SDs, ranging from 0.60 (mean 4.60) for PEU7 to 1.42 (mean 3.81) for PEU2, suggested a variation in the perceptions of ease of use, with the greatest variation in responses relating to the intellectual effort required (PEU2). The skewness for all PEU statements was negative, ranging from –0.99 to –1.99. This indicated a tendency among the physicians to rate the ease of use of teleconsultation systems highly, with a pronounced leaning toward agreement for most statements, particularly PEU7. The kurtosis values for the PEU variables were varied, with some (eg, PEU7) indicating a highly peaked distribution (7.13), which suggests that responses were more consistently clustered around the higher end of the scale. Overall, the data indicated that physicians find teleconsultation systems relatively easy to use. The high mean scores across all PEU items reflected a positive perception of the teleconsultation system’s usability. The variation in SDs, especially the higher deviation for PEU2, suggested that although using the teleconsultation system is generally perceived as easy to use, there are aspects, such as the intellectual effort required, where opinions vary more widely. The pronounced negative skewness, especially for items such as PEU7, underlined a strong agreement in the ease of integrating and using external systems, which might be due to prior familiarity and necessity in clinical practice. The high kurtosis value for PEU7 pointed to a strong consensus among the respondents about the ease of this particular aspect of teleconsultation systems. The physicians generally found teleconsultation systems to be user friendly, with some areas, such as integration with external systems, being particularly well received. Around 253-325 (70.1%-90%) respondents rated the questions in the PEU group positively. However, there was notable variability in perceptions regarding the intellectual effort required, suggesting areas for potential improvement or further training.

The INT variable reflects physicians’ intentions and willingness to continue using teleconsultation services in the future. The mean scores for INT1, INT3, INT4, and INT5 were 4.22 (SD 0.89), 3.99 (SD 1.01), 3.87 (SD 1.12), and 4.06 (SD 0.94), respectively. These scores, being close to or above 4 on a 5-point scale, suggested a generally positive inclination among the physicians toward the continued use of teleconsultations. The SDs for INT1, INT3, INT4, and INT5 indicated a moderate level of variation in responses. This variation signified that although there is a general trend of positive intention, individual physicians’ perspectives on the future use of teleconsultations vary. The skewness values for these variables ranged from –0.97 to –1.20, which are negative. This negative skewness indicated a tendency among respondents to agree with the statements related to INT, suggesting that a larger proportion of physicians are inclined to continue using these services. The kurtosis values for INT1 (1.26), INT3 (0.46), INT4 (0.22), and INT5 (0.79) showed a relatively normal to slightly peaked distribution. This indicated a consistent pattern in physicians’ responses, with a tendency toward agreement on the future use of teleconsultations. The data indicated a positive attitude among physicians toward continuing the use of teleconsultations. The inclination to add video consultations to telephone conversations and to use teleconsultations for patient diagnosis and collaboration with other physicians was evident. The moderate spread in responses, however, pointed to some differences in enthusiasm or confidence about teleconsultations among individual physicians. These differences could be attributed to factors such as personal experience, familiarity with technology, or specific demands of their medical practice. Around 253-289 (70.1%-80%) of respondents intend to use teleconsulting in the future.

The Q variable was rated by physicians as the lowest level of all dimensions. The mean scores ranged from 3.28 (SD 1.26) to 3.73 (SD 1.06). The spread of responses was not even. The mean scores for Q1, Q2, and Q3 were 3.73 (SD 1.06), 3.43 (SD 1.19), and 3.28 (SD 1.26), respectively. These scores indicated a moderate level of agreement among physicians regarding Q, with some variability in the perception of different aspects of Q. The SDs for Q1-Q3 suggested a significant spread in responses. This indicated a varied perception of Q among the physicians, reflecting diverse experiences and expectations regarding teleconsultations. The skewness values for Q1 (–0.82), Q2 (–0.55), and Q3 (–0.27) were negative, implying a tendency for responses to lean toward agreeing with the statements about Q, although this tendency was less pronounced compared to other variables, such as SAT or PU. The kurtosis values for Q1 (–0.01), Q2 (–0.82), and Q3 (–1.18) suggested a relatively flat distribution, particularly for Q2 and Q3. This flatness indicated that the responses were more evenly spread across the scale, reflecting a wide range of opinions on the quality of work. The data suggested that although there is a general trend of moderate satisfaction with Q, there is considerable variation in how physicians perceive this Q. This variability could be influenced by different factors, such as the type of teleconsultation services used, the technological infrastructure in place, and the specific needs of the patient population being served. The more even distribution of responses, especially for Q2 and Q3, indicated that opinions on Q are diverse. This diversity might reflect the complexity of evaluating health care quality in a remote setting, where factors such as patient interaction, diagnostic accuracy, and treatment effectiveness play a crucial role. Only 199 (55.1%) respondents felt that teleconsultations are suitable for holistic patient care, 130 (36%) felt that remote consultations do not allow for comprehensive care, 108 (29.9%) felt that the Q by both methods is not the same, and 224 (62%) believed that the Q using remote visits is similar to that of visits in the clinic. Most physicians (n=260, 72%) agreed with the statement that teleconsultations improve Q.

The SAT variable offers valuable insights into physicians’ contentment and approval levels regarding the use of teleconsultations. Physicians positively rated statements regarding SAT. The mean scores for the 4 statements of the SAT variable (SAT1, SAT2, SAT3, and SAT4) were 3.55 (SD 1.16), 4.13 (SD 0.88), 4.00 (SD 0.99), and 3.91 (SD 1.01), respectively. These scores, hovering around or above 4 on a 5-point scale, indicated a general trend of SAT among physicians with teleconsultation services. The SDs for SAT1-SAT4 suggested a moderate level of variation in responses. This indicated that although there is an overall sense of SAT, there are differences in individual experiences and perceptions regarding teleconsultation services. The skewness values for these variables ranged from –0.62 to –1.14, which are negative. This negative skewness implied a leaning toward higher SAT ratings among the respondents, indicating that more physicians agree with the positive aspects of teleconsultations. The kurtosis values for SAT1 (–0.52), SAT2 (1.51), SAT3 (0.82), and SAT4 (0.78) suggested a mixed distribution pattern. Although SAT1 indicated a relatively normal distribution, SAT2 showed a more peaked distribution, suggesting more consistent high ratings among physicians. The data suggested that physicians are generally satisfied with teleconsultation services, as indicated by the mean scores leaning toward the higher end of the scale. The moderate variation in responses indicated differing levels of SAT, which may be influenced by individual experiences, technological proficiency, or specific needs in their practice. The skewness toward higher SAT ratings suggested that a larger proportion of physicians find teleconsultations to be a convenient and effective medium for providing health care services.

Descriptive statistics of all model variables are presented in Table 4, and distributions of the responses are presented in Table 5.

**Table 4:** Descriptive statistics of model variables.

	Mean (SD)	Variance	Skewness	Kurtosis
PU1^a^	4.36 (0.94)	0.87	–1.73	2.77
PU2	4.04 (1.03)	1.06	–1.25	1.07
PU3	3.85 (1.18)	1.38	–0.91	–0.14
PU4	4.24 (0.87)	0.75	–1.34	1.93
PU5	3.89 (1.14)	1.30	–0.81	–0.38
PU6	4.03 (1.01)	1.01	–1.16	0.99
PEU1^b^	4.28 (0.83)	0.70	–1.34	1.88
PEU2	3.81 (1.42)	2.02	–0.99	–0.47
PEU3	4.51 (0.67)	0.45	–1.82	5.27
PEU4	4.06 (1.04)	1.08	–1.27	1.14
PEU5	4.19 (0.88)	0.77	–1.63	3.44
PEU6	4.42 (0.69)	0.48	–1.48	3.82
PEU7	4.60 (0.60)	0.36	–1.99	7.13
INT1^c^	4.22 (0.89)	0.80	–1.20	1.26
INT3	3.99 (1.01)	1.01	–0.97	0.46
INT4	3.87 (1.12)	1.25	–0.97	0.22
INT5	4.06 (0.94)	0.88	–1.04	0.79
Q1^d^	3.73 (1.06)	1.13	–0.82	–0.01
Q2	3.43 (1.19)	1.42	–0.55	–0.82
Q3	3.28 (1.26)	1.58	–0.27	–1.18
SAT1^e^	3.55 (1.16)	1.35	–0.62	–0.52
SAT2	4.13 (0.88)	0.78	–1.14	1.51
SAT3	4.00 (0.99)	0.98	–1.02	0.82
SAT4	3.91 (1.01)	1.02	–1.07	0.78

^a^PU: perceived usefulness.

^b^PEU: perceived ease of use.

^c^INT: intention to use teleconsultation.

^d^Q: quality of work.

^e^SAT: satisfaction.

**Table 5:** Participant (N=361) response distributions.

Likert scale response	I do not agree, n (%)	I do not agree somewhat, n (%)	I neither agree nor disagree, n (%)	I agree somewhat, n (%)	I agree, n (%)
PU1^a^	7 (1.9)	19 (5.3)	17 (4.7)	113 (31.3)	205 (56.8)
PU2	11 (3.0)	32 (8.9)	22 (6.1)	162 (44.9)	134 (37.1)
PU3	18 (5.0)	45 (12.5)	37 (10.2)	134 (37.1)	127 (35.2)
PU4	4 (1.1)	17 (4.7)	27 (7.5)	152 (42.1)	161 (44.6)
PU5	10 (2.8)	51 (14.1)	43 (11.9)	123 (34.1)	134 (37.1)
PU6	10 (2.8)	27 (7.5)	36 (10.0)	157 (43.5)	131 (36.3)
PEU1^b^	2 (0.6)	19 (5.3)	20 (5.5)	156 (43.2)	164 (45.4)
PEU2	48 (13.3)	35 (9.7)	11 (3.0)	111 (30.7)	156 (43.2)
PEU3	2 (0.6)	6 (1.7)	6 (1.7)	138 (38.2)	209 (57.9)
PEU4	12 (3.3)	29 (8.0)	25 (6.9)	154 (42.7)	141 (39.1)
PEU5	9 (2.5)	14 (3.9)	14 (3.9)	186 (51.5)	138 (38.2)
PEU6	2 (0.6)	6 (1.7)	12 (3.3)	160 (44.3)	181 (50.1)
PEU7	2 (0.6)	2 (0.6)	4 (1.1)	123 (34.1)	230 (63.7)
INT1^c^	4 (1.1)	16 (4.4)	41 (11.4)	136 (37.7)	164 (45.4)
INT3	8 (2.2)	28 (7.8)	52 (14.4)	145 (40.2)	128 (35.5)
INT4	17 (4.7)	35 (9.7)	45 (12.5)	146 (40.4)	118 (32.7)
INT5	5 (1.4)	25 (6.9)	43 (11.9)	157 (43.5)	131 (36.3)
Q1^d^	13 (3.6)	47 (13.0)	47 (13.0)	172 (47.6)	82 (22.7)
Q2	25 (6.9)	78 (21.6)	33 (9.1)	167 (46.3)	58 (16.1)
Q3	29 (8.0)	99 (27.4)	36 (10.0)	136 (37.7)	61 (16.9)
SAT1^e^	23 (6.4)	55 (15.2)	60 (16.6)	148 (41.0)	75 (20.8)
SAT2	6 (1.7)	12 (3.3)	48 (13.3)	157 (43.5)	138 (38.2)
SAT3	10 (2.8)	19 (5.3)	59 (16.3)	146 (40.4)	127 (35.2)
SAT4	11 (3.0)	33 (9.1)	36 (10.0)	178 (49.3)	103 (28.5)

^a^PU: perceived usefulness.

^b^PEU: perceived ease of use.

^c^INT: intention to use teleconsultation.

^d^Q: quality of work.

^e^SAT: satisfaction.

### Structural Equation Modeling

**Figure 3:**
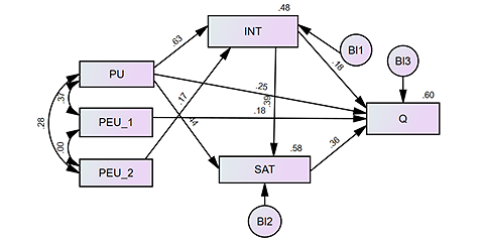
Structural model presenting standardized estimates. BI: measurement error; INT: intention to use teleconsultation; PEU: perceived ease of use; PU: perceived usefulness; Q: quality of work; SAT: satisfaction.

Based on the factors extracted in EFA, a structural model was developed (Figure 3). The model demonstrated excellent fit with the data, as indicated by indices such as the chi-square-to-degrees-of-freedom index: PCMIN/DF=0.91 (<5 is acceptable), comparative fit index (CFI)=1 (>0.9 is acceptable), goodness-of-fit index (GFI)=0.99 (>0.9 is acceptable), and root mean square error of approximation (RMSEA)=0 (<0.08 is acceptable). These values suggested that our model is robust and accurately represents the observed data.

In assessing the relationships between key constructs in our teleconsultation acceptance model, we used maximum likelihood estimates to derive regression weights (Table 6). Our analysis revealed significant relationships between the constructs, as evidenced by the *P* values and critical ratios (CRs) in the regression weights. The influence of PU on INT was strong (estimate=0.63, CR=15.84, *P*<.001), suggesting that physicians’ PU of teleconsultations significantly predicts their INT. PEU_2 also positively influenced INT (estimate=0.17, CR=4.31, *P*<.001), albeit to a lesser extent than PU. Both PU (estimate=0.44, CR=9.53, *P*<.001) and INT (estimate=0.4, CR=8.57, *P*<.001) significantly predicted SAT, indicating that PU and INT are crucial determinants of SAT with teleconsultations. Q was positively influenced by INT (estimate=0.179, CR=3.64, *P*<.001), PU (estimate=0.246, CR=4.79, *P*<.001), PEU_1 (estimate=0.18, CR=4.93, *P*<.001), and SAT (estimate=0.357, CR=6.97, *P*<.001), highlighting a multifaceted impact on Q. Since on each path of the model, the values of the regression parameters had *P*<.05, it can be said that at a significance level of =.05, all the direct dependencies of the model are significant, and hence, H1-H8 are supported.

Standardized regression weights underscore the relative strength of these relationships in standardized format, which is particularly useful for comparing the effects across different predictors within our model. A significant positive covariance was observed between PU and PEU_1 (estimate=0.37, CR=6.52, *P*<.001) and between PU and PEU_2 (estimate=0.28, CR=5.05, *P*<.001), suggesting that PU and PEU are interrelated constructs. However, no significant correlation was found between PEU_1 and PEU_2 (estimate=0, *P*=.99), indicating these aspects of PEU may independently influence the model. The squared multiple correlations for INT (0.48), SAT (0.58), and Q (0.6) indicate a substantial proportion of variance in these endogenous variables, explained by their respective predictors.

Our findings provide strong empirical support for the hypothesized relationships within TAM. The significant paths between constructs such as PU, PEU, INT, SAT, and Q highlight the multifaceted nature of teleconsultation acceptance among physicians.

These results underscore the importance of both PU and PEU in influencing INT and SAT, which, in turn, impact Q.

**Table 6:** Standardized regression weights of direct model paths.

Model paths	Estimate	*P* value
PU^a^→INT^b^	0.626	<.001
PEU^c^_2→INT	0.17	<.001
PU→SAT^d^	0.439	<.001
INT→SAT	0.395	<.001
INT→Q^e^	0.179	<.001
PU→Q	0.246	<.001
PEU_1→Q	0.176	<.001
SAT→Q	0.357	<.001

^a^PU: perceived usefulness.

^b^INT: intention to use teleconsultation.

^c^PEU: perceived ease of use.

^d^SAT: satisfaction.

^e^Q: quality of work.

### Mediation Analysis

In addition to the analysis of the model’s direct paths, a mediation analysis was conducted. The values of regression factors for indirect model paths were determined (Table 7). The mediating effects within the structural model for the teleconsultation research provide insights into the indirect pathways through which independent variables influence dependent variables. PU had a substantial indirect effect on SAT through INT, with an estimate of 0.25 (*P*<.001). This suggests that the PU of teleconsultation systems significantly influences SAT, mediated by INT. Additionally, PU indirectly affected Q through both INT (estimate=0.112, *P*<.002) and SAT (estimate=0.16, *P*<.001). These paths indicate that PU leads to higher Q, as it influences INT and SAT with it. PEU_2 indirectly influenced SAT via INT, with an estimate of 0.07 (*P*<.001). This effect signifies that the PEU contributes to SAT through INT. For Q, PEU_2 had an indirect effect through the mediation of INT (estimate=0.03, *P*<.001) and SAT (estimate=0.02, *P*<.001). These findings imply that PEU of teleconsultations not only impacts INT but also enhances the Q delivered through SAT. The path from INT to Q mediated by SAT was significant, with an estimate of 0.14 (*P*<.001), indicating that INT contributes to Q, as mediated by SAT levels. The bootstrap 95% CIs for these indirect effects reinforced their significance, as they did not include 0, and the *P* values were well below the .001 threshold, indicating robustness in these mediating relationships. The mediating effects elucidate the important role of INT and SAT with teleconsultation services in enhancing the PU and PEU.

Our findings from the SEM analysis corroborate several key hypotheses concerning direct, indirect, and mediating effects within the teleconsultation context. In accordance with H1, PU exhibited a significant direct effect on INT. Supporting H2, PU was also found to directly affect Q. In line with H3, a direct effect of PU on SAT was substantiated. PEU_1’s direct effect on Q affirmed H4, demonstrating the technical influence of PEU on Q. PEU_2 was confirmed to directly impact INT, lending credence to H5. H6, positing a direct effect of INT on SAT, was also validated. Similarly, H7 was supported, with INT having a direct influence on Q. SAT was found to directly affect Q, confirming H8. The indirect influence of PU on Q through INT, posited in H9, was also substantiated. PU was found to indirectly affect SAT through INT, supporting H10. Moreover, the hypothesized indirect effect of PU on Q via the mediating roles of INT and SAT, as stated in H11, was validated. PEU_2’s indirect impact on Q through INT, as hypothesized in H12, was confirmed. The indirect effect of PEU_2 on SAT via INT, detailed in H13, was likewise corroborated (Table 8).

Since on each path of the model, the values of the regression parameters had *P*<.05, it can be said that at a significance level of =.05, all the indirect dependencies of the model are significant, and hence, H9-H14 are supported.

**Table 7:** Standardized regression weights of indirect model paths.

Indirect model paths	Estimate	*P* value	Significance
PU^a^→INT^b^→SAT^c^	0.247	<.001	Significant
PU→INT→SAT→Q^d^	0.088	<.001	Significant
PU→INT→Q	0.112	<.002	Significant
PU→SAT→Q	0.157	<.001	Significant
PEU^e^_2→INT→SAT	0.067	<.001	Significant
PEU_2→INT→SAT→Q	0.024	<.001	Significant
PEU_2→INT→Q	0.03	<.001	Significant
INT→SAT→Q	0.141	<.001	Significant

^a^PU: perceived usefulness.

^b^INT: intention to use teleconsultation.

^c^SAT: satisfaction.

^d^Q: quality of work.

^e^PEU: perceived ease of use.

**Table 8:** Corroboration of hypotheses concerning direct, indirect, and mediating effects.

Hypothesis (H)	Description	Supported (yes/no)
H1	PU^a^ has a direct effect on INT^b^.	Yes
H2	PEU^c^ has a direct effect on INT.	Yes
H3	PU has a direct effect on SAT^d^.	Yes
H4	INT has a direct effect on SAT.	Yes
H5	PU has a direct effect on Q^e^.	Yes
H6	PEU has a direct effect on Q.	Yes
H7	INT has a direct effect on Q.	Yes
H8	SAT has a direct effect on Q.	Yes
H9	PU has an indirect effect on Q through INT.	Yes
H10	PU has an indirect effect on SAT through INT.	Yes
H11	PU has an indirect effect on Q through INT and SAT.	Yes
H12	PEU_2 has an indirect effect on Q through INT.	Yes
H13	PEU_2 has an indirect effect on SAT through INT.	Yes
H14	PEU_2 has an indirect effect on Q through INT and SAT.	Yes

^a^PU: perceived usefulness.

^b^INT: intention to use teleconsultation.

^c^PEU: perceived ease of use.

^d^SAT: satisfaction.

^e^Q: quality of work.

## Discussion

### Principal Findings

This study evaluated Polish physicians’ acceptance of teleconsultations during the COVID-19 pandemic in Poland. Most of the physicians positively assessed the PU of teleconsultations. The majority of physicians believed that their work during the COVID-19 pandemic would have been difficult without teleconsultations (88%) and that teleconsultations turned out to be a useful system enabling medical care (87%). The least number of physicians said that teleconsultations save time (61%) and improve performance (72%). Physicians are willing to use new technologies if they do not require additional time and effort, which is in line with other studies [25]. Similar results regarding the usefulness of teleconsultations during a pandemic and the ease of using them were obtained in a cross-sectional study conducted in 2020 in one of the Romanian counties using a questionnaire that assessed, among other things, the perception of teleconsultations by physicians. The study showed a positive perception of telemedicine by Romanian physicians. However, the researchers also highlighted the cons of teleconsultations, such as the time-consuming process, fear of making medical errors remotely, and communication difficulties on the part of patients [57]. The time-consuming nature of teleconsultations has also been confirmed in Great Britain; British physicians reported on time-consuming daily phone calls, emails, and complex electronic medical record protocols [58].

The PEU was also highly rated (average above 4). Most of the surveyed physicians (97%) declared that they know how to connect to external systems during teleconsultations. Using teleconsultations was understandable for most of the respondents (96%), and most of them (94%) could easily prepare all necessary documents (prescriptions, sick leave, referrals for tests, etc) during the teleconsultations. Our results confirm those of other studies according to which the teleconsultations are simple and support physicians’ responsibility in their work and medical decisions [30,59].

Polish physicians also positively assessed the future of teleconsultations and declared their intent to this form of work with patients (83%) and other physicians to agree on the diagnosis (73%). According to the majority of respondents (79%), remote monitoring of patients’ health would improve the performance of teleconsultations. In the future, they (75%) would also willingly use video visits to facilitate contact and diagnosis of patients. Similar findings were obtained in a Romanian study, in which physicians concluded that telemedicine should be used continuously, not just during the COVID-19 pandemic. Most physicians (91.1%) considered it necessary to provide care using telemedicine after the pandemic [57]. In addition, in Brazil, most physicians want to continue remote care and demand regulations on the use of telemedicine that would allow the extension of remote services [60].

Teleconsultation became popular during the COVID-19 pandemic, and now, there are expectations that it will become a permanent part of the health care system [61]. The development and integration of ICT in health care delivery have great potential for patients, providers, and payers in future health care systems [62].

Polish physicians positively assessed SAT and felt comfortable giving the system a high SAT score. The mean value of responses for all statements regarding SAT was approximately 4. Only the statement regarding the identity of remote and traditional visits was rated lower. Only 62% of physicians believed that both forms of medical consultations are equivalent, and the average for this dimension was 3.55. This is probably due to the influence of teleconsultations on Q, which Polish physicians assessed as the lowest of all the dimensions of TAM. The average response ranged from 3.28 to 3.73. Only 55.1% of respondents stated that teleconsultation is suitable for holistic patient care, and 36% stated that teleconsultation does not allow for comprehensive care. The literature also emphasizes that teleconsultations will never replace F2F meetings. The large-scale and urgent introduction of teleconsultation into our practice is likely to be redefined in the post–COVID-19 era [7]. Another opinion is that teleconsultation is not inferior to personal visits to the office in terms of the preferences and satisfaction of patients and physicians. It should, therefore, be an effective complement to F2F office visits as a mechanism for segregation and long-term continuity of care [63].

### Comparison With Previous Studies

This is the first such study conducted on SAT and Q in PHCs. SAT and Q have already been studied in other medical specialties (eg, urology, dermatology, psychiatry, and oncology). In a study conducted among dermatologists, almost all categories regarding SAT with remote dermatological teleconsultations were rated at about 9 on a 0-10 scale [64]. Schubert et al [65] assessed SAT with teleconsultations in psychiatry, which turned out to be at a high level. Providers were satisfied with telepsychiatry, and both believed that telepsychiatry provides patients with better access to care. Urological teleconsultation introduced quickly during the COVID-19 closure has achieved a high level of satisfaction among both patients and physicians [7]. Physicians are interested in using telemedicine tools that increase improved access to health and differentiate their clinical practice [66]. Telemedicine benefits all physicians’ patients by increasing access to health care services and remotely managing elderly people with chronic conditions [67]. Therefore, the findings of our research are in accordance with other studies documenting the openness of physicians to the use of teleconsultations in providing health services to patients [68].

However, teleconsultations have limitations regarding the uncertainty caused by the inability to physically check the patient’s health condition, and this is something physicians should be aware of [69]. Studies so far show that almost two-thirds of physicians report uncertainty about the correctness of a diagnosis made with telemedicine, and only one-fourth have confidence in making remote decisions [57]. Teleconsultations will never fully replace a personal visit, due to the inability to check the physical symptoms of the disease and the lack of nonverbal signals expressing trust and empathy during remote contact.

SEM results substantiate the significant influence of PU on INT, SAT with this technology, and Q. A possible reason for this may be the availability and effectiveness of teleconsultation, the time saving in this system, and Q. Thus, a positive effect from PU will result in better SAT with teleconsultations and INT. This result is in line with other studies conducted in the field of telemedicine [30,70-73].

The medical PEU from the point of view of handling medical data (PEU_2) has a minor but significant impact on INT. Notably, the technical PEU_1 has a significant impact on Q. The easier it is to use teleconsultation, the better physicians are at assessing Q. The less effort users put in to handle medical data, the more positive their INT to use the system. This is in line with other studies, showing that the acceptance of telemedicine is greater when it provides faster health care, cost savings, better documentation, and time savings [52]. However, a study conducted in the United States found that the role of the influence of PEU on INT is insignificant. The study focused on pediatricians’ INT to use of online health apps. The reason for this could be the longer contact of physicians with telemedicine technology [64]. Another explanation is that for highly competent physicians, the effect of PEU on INT is of little importance [74].

PU and PEU are considered the main determinants that directly explain the intent to use (“accept”) a new technology [75]. In this study, we, therefore, confirmed the hypotheses that the constructs described in the traditional TAM are appropriate for measuring the intent of physicians to use teleconsultations.

INT emerged as a factor influencing SAT and Q and a pivotal mediator linking PU with both SAT and Q, thus underscoring the importance of intentions in the acceptance and effective use of telemedicine. This finding is in harmony with the existing literature that emphasizes the mediating role of INT in the context of technology acceptance [26]. When physicians believe that using teleconsultation will be effortless and useful, their attitude and INT will improve. This system, with less effort, encourages physicians to use teleconsultations and improves SAT and Q [31,70,76,77]. Therefore, the condition for the implementation of telemedicine technologies should be ensuring its understanding by health care providers in order to gain their acceptance and ensure the use of these technologies in the future [78]. SAT also turned out to be a significant mediator between PU, PEU, and Q. Our research, therefore, showed that the main elements of TAM viewed as PU and PEU have a significant impact on INT, which has been confirmed in other studies [30,70-73,79-82].

The model reaffirms the significant direct and indirect roles of PU and PEU in shaping INT, SAT, and Q. These findings contribute to the extant literature on teleconsultation acceptance and underscore the nuanced factors that influence the acceptance and satisfaction of teleconsultation services among physicians.

### Implications

When planning a new teleconsultation system, PHC facilities should be able to predict whether the new system will be acceptable and satisfactory for medical staff, investigate the reasons why the planned system may not be fully acceptable, and then take action to increase the system’s acceptance. The results of this study show that the PU of a system is a key determinant of medical professionals’ INT. Therefore, before introducing a new system to PHC facilities, managers of these facilities can increase the acceptance of the system by involving medical personnel in the implementation process, assessing the medical personnel’s perception of the system (PU and PEU) and taking appropriate actions based on this assessment. Training should also be provided to medical staff to highlight the effectiveness and usefulness of teleconsultation in PHCs. Information and training sessions should primarily focus on how teleconsultation can help improve the quality of PHCs.

Intention as an intermediary variable has a significant and positive impact on users’ SAT with teleconsultation and its Q. To increase the expected results in the Q of teleconsultations, the teleconsultation system should be useful for health checking, improving the quality of life, and increasing the capacity for self-care. To increase PEU, the teleconsultation system should be clear, understandable, easy to learn, easy to implement, and easy to perform health checks with. To increase the perceived utility, the teleconsultation system should positively influence the treatment plan, provide more comprehensive care services, and efficiently diagnose and efficiently plan and precisely monitor the patient’s condition. The system should make physicians willing to use it to increase their INT. All this will contribute to greater SAT of physicians with their work and better quality of care [83].

This study is a contribution to the field of teleconsultation acceptance research. The modified TAM and its psychometric properties verified in this study can be used as a research framework to understand the acceptance of teleconsultation, especially in the population of PHC workers. The model can also be used for future TAM research in a variety of contexts in identifying, explaining, and predicting the intention of PHC professionals to use teleconsultation. Therefore, it is highly recommended to replicate this study in different environments to generalize the results across domains.

### Limitations

In the discussion of research findings, it is crucial to acknowledge the limitations of this study to provide a comprehensive understanding of its context and implications. Although the study offers valuable insights into the factors influencing the acceptance and SAT of medical professionals with teleconsultation systems, several limitations must be considered.

The study was conducted across a specific number of PHC facilities in Poland. Although efforts were made to ensure a representative sample of PHC facilities, the findings might not fully encapsulate the diverse range of experiences and perceptions of all medical professionals nationwide. Regional variations, different health care settings, and varying levels of teleconsultation acceptance could influence SAT and acceptance levels.

The cross-sectional nature of the survey limits our ability to infer causality or changes over time. Longitudinal studies would be required to understand how perceptions and SAT with teleconsultation evolve as users gain more experience and as the technology itself advances.

The reliance on self-reported data can introduce biases, such as social desirability or recall bias. Participants’ responses might not accurately reflect their true experiences or feelings toward teleconsultation.

Although the study focused on PU and PEU, other factors could influence SAT and INT. These might include individual technological proficiency, prior experiences with teleconsultation, or organizational support, which were not extensively explored in this study.

The study primarily addressed teleconsultation in PHC settings. The findings might not be generalizable to other forms of teleconsultation or to specialists’ use of teleconsultation, where different factors could be more influential. The study also did not deeply explore the technological and operational constraints that might impact the effectiveness and user SAT with teleconsultation systems, such as system reliability, user interface design, and integration with existing health information systems.

The study was conducted during a period potentially influenced by the COVID-19 pandemic, which might have affected attitudes toward teleconsultation. The urgency and necessity of teleconsultation during the pandemic might not reflect standard operational conditions.

By addressing these limitations, future research can build upon the findings to develop a more nuanced understanding of the factors influencing the successful implementation and adoption of teleconsultation systems in various health care settings. In the forthcoming models, we also intend to include constructs such as compatibility, self-efficacy, social norms, perceived behavioral control [25,84], social interaction, invasiveness, and relevance [85].

### Conclusion

After the outbreak of the COVID-19 pandemic, there was a dynamic development of teleconsultations in PHCs in Poland. Therefore, we conducted satisfaction surveys of Polish physicians based on a modified TAM, which we extended with new constructs, including physicians’ SAT and Q, considering INT as a mediating variable. The tool developed for this model was verified in terms of psychometric properties. Therefore, it has the potential to be used in both research and practice, especially to assess the SAT and Q of PHC physicians who use teleconsultations in Poland.

The findings highlight significant relationships between PEU, PU, INT, and physicians’ SAT with teleconsultation and their Q. The study showed that the PEU and PU of teleconsultations are predictive determinants of the acceptance of teleconsultation, which in turn influences physicians’ SAT and Q.

Identification of the most important factors influencing physicians’ SAT and Q can provide important information to managers of PHC facilities and help them make the right decisions. This study provides information for the strategies of PHCs and policy makers to accept and encourage the use of teleconsultations in Poland.

## Appendix

Multimedia Appendix 1 Questionnaire statements used in the modified Technology Acceptance Model.

## Abbreviations

CR: critical ratio
EFA: exploratory factor analysis
F2F: face-to-face
ICT: information and communication technology
INT: intention to use teleconsultation
KMO: Kaiser-Meyer-Olkin
NHF: National Health Fund
PEU: perceived ease of use
PHC: primary health care facility
PU: perceived usefulness
Q: quality of work
SAT: satisfaction
SEM: structural equation modeling
TAM: Technology Acceptance Model

## Data Availability

The data sets generated and analyzed during this study are available from the corresponding author upon reasonable request.
