# Supplementary material for: Predictive Factors of Physicians’ Satisfaction and Quality of Work Under Teleconsultation Conditions: Structural Equation Analysis
Source: JMIR Hum Factors. 2024 Jun 10;11:e47810. doi: 10.2196/47810 (PMC11216023; doi:10.2196/47810)
Supplement: Multimedia Appendix 1 [file humanfactors_v11i1e47810_app1.docx]

Appendix 1. Survey statements.

| Dimension | Variable | Survey statement |
| --- | --- | --- |
| Perceived Usefulness  PU | PU1 | My work during a pandemic would be difficult without teleconsultations |
|  | PU2 | Teleconsultations meet my at work |
|  | PU3 | Teleconsultations increase the efficiency of my work |
|  | PU4 | In general I find the teleconsultations a useful system in my work |
|  | PU5 | Teleconsultations save my time |
|  | PU6 | Teleconsultations make my work easier |
| Perceived Ease of Use  PEU | PEU1 | Using a teleconsultations system is easy |
|  | PEU2 | Using a teleconsultations system does not require too much intellectual effort |
|  | PEU3 | Using the teleconsultations system is understandable to me |
|  | PEU4 | Using the teleconsultations system, I can do everything I want |
|  | PEU5 | I can easily access information about the patient while using the teleconsultations system |
|  | PEU6 | I can easily prepare all necessary documents (prescriptions, sick leave, referrals for tests, etc.) during the teleconsultations |
|  | PEU7 | I know how to connect to external systems (e.g. ZUS) during teleconsultations |
| Intention to use teleconsultations  INT | INT1 | If possible, I intend to use the teleconsultations system in the future |
|  | INT3 | The use of video visits would facilitate my contact with and diagnosis of patients |
|  | INT4 | I would be happy to use a teleconsultations system to agree on a diagnosis with other doctors |
|  | INT5 | Remote monitoring of a patient's condition would improve the effectiveness of Telemedicine |
| Quality of work  Q | Q1 | Teleconsultations improve the quality of my work |
|  | Q2 | The quality of work with the use of the teleconsultations system is similar to that of a traditional visit |
|  | Q3 | The teleconsultations system allows for comprehensive patient care |
| Physicians ' satisfaction  SAT | SAT1 | Teleconsultations are more convenient compared to normal office visits |
|  | SAT2 | Overall, I am satisfied with the use of the teleconsultations |
|  | SAT3 | I would like to use the possibility of teleconsultations again |
|  | SAT4 | I feel comfortable giving advice remotely |
|  | SN2 | People who have influence on my work think that I should use teleconsultations |
|  | SN4 | Medical colleagues think that teleconsultations is worth using |
